# A238 A MULTIDISCIPLINARY APPROACH FOR PANCREATICOBILIARY CANCER WITH EMPHASIS ON TIME TO CARE ACCESS: AN OVERVIEW OF THE EDMONTON PANCREATICOBILIARY INFLAMMATION AND CANCER (EPIC) PROGRAM

**DOI:** 10.1093/jcag/gwae059.238

**Published:** 2025-02-10

**Authors:** N Ashrafinia, M Timmermans, G Buchanan, S Perryman, A Kwan, E Bereznicki, P Mathura, G Sandha

**Affiliations:** University of Alberta Faculty of Medicine & Dentistry, Edmonton, AB, Canada; Alberta Health Services, Edmonton, AB, Canada; Alberta Health Services, Edmonton, AB, Canada; Alberta Health Services, Edmonton, AB, Canada; Alberta Health Services, Edmonton, AB, Canada; Alberta Health Services, Edmonton, AB, Canada; University of Alberta Faculty of Medicine & Dentistry, Edmonton, AB, Canada; University of Alberta Faculty of Medicine & Dentistry, Edmonton, AB, Canada

## Abstract

**Background:**

Persistent challenges in pancreaticobiliary (PB) cancer management continue to have a significant impact on patients. Prolonged access to care, for diagnosis and treatment, prompted the launch of a multidisciplinary approach in 07/2023 to provide timely access to patient-centred points of care, and to improve overall quality of life (QOL).

**Aims:**

To evaluate the outcomes of the multidisciplinary EPIC program, with a focus on access to care timelines established in a collaborative care pathway developed for this program.

**Methods:**

All consecutive patients with suspected PB cancer presenting to the University of Alberta Hospital between 07/2023 and 08/2024 were referred to the EPIC program. Referrals were triaged by gastroenterology, pancreaticobiliary surgery, and the EPIC nurse navigator (NN) into resectable, partially resectable, and unresectable cases. Endoscopic intervention for biopsy and/or stent placement was scheduled as necessary, and referral to the local cancer cantre when indicated. Appropriate referrals were made to palliative and dietary services. The NN ensured timely referral and scheduling in accordance with the care pathway timelines and served as the primary point of contact for patients throughout their journey. Data was entered into the REDCap database. Descriptive statistics were performed.

**Results:**

A total of 254 patients (152 male, 102 female) with a median age of 68±11 years were referred. Presenting symptoms were abdominal/back pain (72%), weight loss (47%), jaundice (36%), nausea/vomiting (18%), pruritus (9%), coincidental finding (6%), new-onset diabetes (6%), and fever (1%). The site of cancer was pancreas (81%), bile duct (13%), ampulla (2%), and others 3%. The median times to access are shown in the table and figure. The 6- and 12-month survival for resectable pancreatic cancer was 93% and 82%, respectively, whereas for unresectable pancreatic cancer was 44% and 37%, respectively.

**Conclusions:**

A NN-led, patient-centred care model is crucial for timely access to care in PB cancer. Improved coordination of care and overall improvement in patient QOL were demonstrated with the initiation of this program.

TIME TO CARE ACCESS IN DAYS, median ± SD

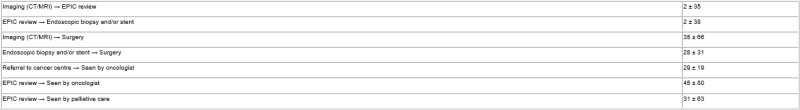

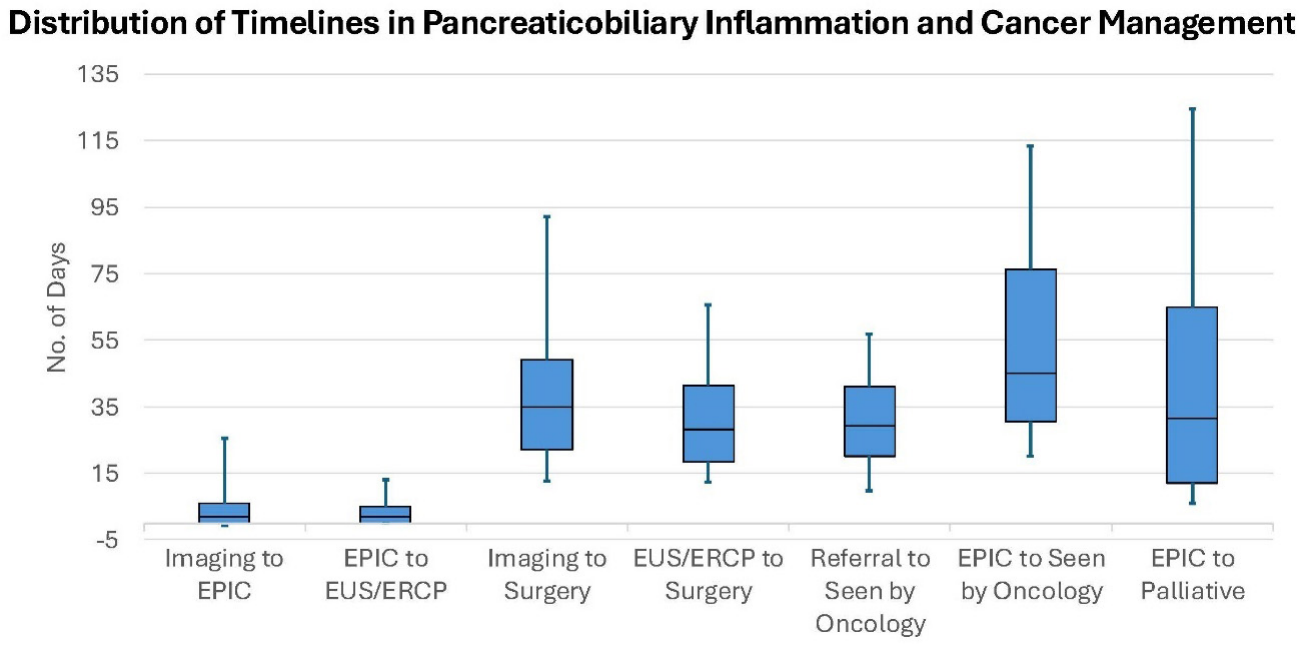

**Funding Agencies:**

None

